# Parents' Experience and Satisfaction in Neonatal Intensive Care Units in Ethiopia: A Multicenter Cross-Sectional Study Using an Adapted Version of EMPATHIC-N

**DOI:** 10.3389/fped.2021.738863

**Published:** 2021-10-08

**Authors:** Berhanu Gulo, Laura Miglierina, Francesca Tognon, Silvia Panunzi, Ademe Tsegaye, Tina Asnake, Fabio Manenti, Immacolata Dall'Oglio

**Affiliations:** ^1^Doctors With Africa CUAMM, Addis Ababa, Ethiopia; ^2^Doctors With Africa CUAMM, Padua, Italy; ^3^Department of Women's and Children's Health, University of Padua, Padua, Italy; ^4^Unit of Epidemiology and Medical Statistics, Department of Diagnostics and Public Health, University of Verona, Verona, Italy; ^5^Federal Ministry of Health, Addis Ababa, Ethiopia; ^6^Professional Development, Continuous Education and Research Service, Bambino Gesù Children's Hospital IRCCS, Rome, Italy

**Keywords:** parents, satisfaction, neonatal intensive care unit, validation study, surveys and questionnaires, EMPATHIC, multicenter study, Ethiopia

## Abstract

**Background:** In neonatal intensive care units (NICU) setting, parents' experience and satisfaction permit to evaluate clinical practice and improve the care of infants and parents. Little is known about this topic in low resource settings. The aim of this study was to (1) translate, adapt and validate the EMpowerment of PArents in THe Intensive Care-Neonatology (EMPHATIC-N) questionnaire in two languages in Ethiopia (2) explore parents' satisfaction with the care received in the NICUs in three hospitals; and, (3) explore socio-demographic characteristics and level of the NICU influence on the EMPATHIC-N domains.

**Methods:** This was a cross-sectional multicenter study. Participants were recruited from three different NICUs in Ethiopia upon discharge. We reduced the original EMPATHIC-N instrument to 38 items, culturally adapted and validated it in two local languages. Confirmatory Factor Analysis (CFA) was applied to verify the factor structure of the questionnaire, investigating the relationship between items and the five latent domains. Single item scores and the aggregate scores of the domains were investigated across NICUs and in the sample overall. Differences in the distribution of the domain scores were tested according to socio-demographic participants' characteristics. The scores of four general questions about overall experience and satisfaction were investigated in relation to the participant's characteristics and NICU levels. Qualitative data were collected using four open-ended questions and a synthesis of results was provided.

**Results:** Almost all the parents answered to the questionnaire (92%, *n* = 386). Questionnaire items on satisfaction on average scored more than four. The highest mean scores were obtained for Parental participation (median: 5.17; iqr: 4.67–5.62), while they were lower for Organization/Hospital environment (median: 4.67; iqr:4.33–5.17). Different levels of parent satisfaction were observed across the NICU levels showing a statistically higher satisfaction in level II NICU compared to the other levels. Education, place of residence and length of stay were associated with parental satisfaction and experience.

**Conclusion:** This study validated two Ethiopian versions of the EMPATHIC-N questionnaire to assess parents' experience and satisfaction during their child's stay in the NICU. The differences found across the three levels of NICU suggest the need to further investigate the determinants of satisfaction.

## Introduction

Parental experience is a crucial measure of service quality and could point to ways of improving health care delivery (1, 2). Particularly in the neonatal intensive care unit (NICU) setting, parent satisfaction is one of the quality care indicators mainly concerned with fulfilling parents' positive expectations of the perceived factors of the child's care (1).

Parent involvement and empowerment play an important role in building a family-centered care (FCC) environment (3) with beneficial effects for both infants and parents (4). The attributes of FCC in the NICU have been recognized as participation of the family (in care planning, decision making, and providing care from routine to specialized care), family's respect and dignity, and knowledge transformation (information sharing between healthcare providers and families, complete information sharing according to the family's learning style) (5).

Studies have shown significant associations between parental satisfaction with health care in NICUs and their ability to provide appropriate care for their infants. Generally, higher satisfaction with health care is reported to yield better treatment compliance (1, 3). Interaction with healthcare professionals (HCPs) is crucial for parental experience (6). For health workers, it is important to understand parents' expectations when infants are admitted to the NICU, to meet their needs and concerns and increase their satisfaction, which will promote more appropriate attachment and bonding (7).

The EMpowerment of PArents in THe Intensive Care-Neonatology (EMPATHIC-N) questionnaire was developed in the Netherlands to measure NICU parent satisfaction and experience (3) and has been translated and culturally adapted in several countries (2, 8–10). A similar questionnaire related to the pediatric intensive care unit was culturally translated in South Africa (11). Although parents' experiences in pediatric health services have already been evaluated by other authors in Ethiopia (12, 13), currently a validated instrument to specifically evaluate the NICU context is still not available in that country.

In the last 10 years, the Ethiopian Ministry of Health has worked hard to establish new NICUs in hospitals, and strengthen existing ones through training and continued support for newborn HCPs providers and managers, ensuring the equipment and supplies are in place, and strengthening the facility infrastructure and the referral system (14, 15).

The Ethiopian Federal Ministry of Health (FMOH) is formalizing the guidelines regarding the level of neonatal care services, defining the standards in terms of space, personnel, quantity of NICU supplies and equipment. The draft of this document, shared by the FMOH to the authors, divides NICUs into 3 levels. Level-I NICUs are supposed to have personnel and equipment to perform neonatal resuscitation, evaluate and provide postnatal care for healthy and sick newborn infants, stabilize and provide care for infants born between 35 and 37 weeks of gestation and should be guaranteed by all district hospitals. Level-II NICUs should provide care to infants born after 32 weeks of gestation and weighing more than 1500 g (managing problems caused by physiologic immaturity) and should be found at least in all regional referral hospitals. Specialized teaching hospitals should have a Level-III NICU to provide life support and comprehensive care for extremely high-risk newborn infants and those with complex and critical illnesses (16).

The three levels of complexity imply different structural organizations and different expectations of the population which can greatly influence the parental experience. Investigating these differences can be helpful when planning specific interventions depending on the level of NICU.

This study was conducted within the framework of a 3-year project implemented by Doctors with Africa CUAMM (University College for Aspiring Missionary Doctors) that aimed at improving the quality of NICU services in the three selected hospitals. The project, designed together with the FMOH, started in 2018 and included operational research and surveys on newborn care to generate evidence to enable the FMOH to make informed decisions.

Since sociocultural and economic contexts may have a strong influence on parents' experiences (17), it is important to take into account the peculiarities of the context both during the validation process of the tool and the interpretation of the data.

The complexity of applying a user assessment tool in the Ethiopian context is increased by the many different languages spoken by the population in Ethiopia.

The aim of this study was to (1) translate, adapt and validate the EMPHATIC-N in two spoken languages in Ethiopia (2) explore parents' satisfaction with the care and treatment received in the NICUs in three hospitals and (3) explore socio-demographic characteristics and level of the NICU influence on the EMPATHIC-N domains.

## Materials and Methods

### Design

This is was multi-center cross-sectional quantitative study that measured client satisfaction with NICU services. The study included a qualitative section to explore the parents' answers to the open questions.

### Ethical Issues

The medical research ethics review board of one of the hospitals (an Academic Hospital) and of the Oromia Regional Health Bureau approved the study protocol. Considering the local cultural context (18), a verbal informed consent was obtained from all study participants (19–21). Parents were informed about the study objectives and data were collected anonymously. The interviewer gave the respondents enough information so that they could provide an informed decision on whether to participate in part or in the entire interview. Afterwards, the consent forms were signed by the interviewer as evidence that informed consent had been obtained from the interviewer.

### Setting

The study was conducted in three different types of NICUs in Ethiopia: a Level I NICU with 8 beds run by one General Practitioner and 7 Nurses (4 of them with a 1-month training session for NICU care); a Level II NICU with 6 beds run by a Pediatrician, a Health Officer and 12 Nurses (4 of them with a 1-month training session for NICU care); a Level III NICU with 14 beds run by 2 Neonatologists, 2 Pediatricians, 9 Resident Doctors, 8 Neonatal Nurses, 48 Nurses (12 of them with a 1-month training session on NICU care). The average number of monthly admissions in the first semester of 2019 was 23, 81, and 168 for Level I, II, and III, respectively. Level I and II were located in the Oromia region, while Level III, an Academic Hospital, was located in Addis Abebe, the capital city. Level I and Level III NICUs were located in government hospitals, while Level II NICU was located in a private not-for-profit hospital supported by Doctors with Africa CUAMM. Admission and treatment costs in all the NICUs were free of charge as per national policy.

### Sample

Study participants were all parents whose newborns had been discharged from one of the three NICUs or transferred to another unit during the study period.

Only the parents of the newborns admitted to the NICUs for more than 2 days were included in the study. Parents with multiple births discharged on the same day, received only one satisfaction questionnaire. Parents whose infants died while in the unit were excluded from this study.

If both parents were present, only one of the parents was interviewed, leaving the decision to the couple.

Sample size was determined conservatively by considering 10 respondents for each of the items (22) and a possible 10% non-response rate was calculated, with *N* = 418 parents to be interviewed.

The sample size was divided proportionally across the three hospitals considering the average monthly admissions of newborns. All the eligible respondents were interviewed until the desired sample size was achieved.

### Study Variables and Operational Definition

Socio-demographic variables such as age, sex, educational level (high level of education was considered from secondary schools, high school, grade 9–12 in Ethiopia), occupation (housekeeping or any type of occupation) and place of residence (urban or rural, based on the country's administrative arrangements), were collected from the parents interviewed. Moreover, infants' sex and length of hospital stay were collected from inpatients' clinical records.

Five specific dimensions (Communication-Information and Education, Care & treatment, Organization/ Hospital Environment, Parental participation, and Professional Attitude) related to parent satisfaction were investigated.

A 6-point Likert scale, from one, “not at all satisfied,” to six, “all the time satisfied,” was used to assess the 38 questionnaire items -responses, with higher scores indicating greater parent satisfaction.

General experience outcomes answers (items Q39 and Q40), “Recommend this NICU to anyone” and “Would you come back” were conveniently dichotomized from the above 6-point scale to “Not at all/a little/to some extent” if 1–4 points were scored and to “Most of the time /always” if items were scored 5–6 points. Answers “in some extend/largely satisfied” were included in the first level together with the “not and little satisfied” to have more balanced groups, because almost every parent was answering “very largely/all the time satisfied” to these questions.

Answers about general impression (items Q41 and Q42 were dichotomized conveniently from a 5-point scale into “Very dissatisfied, quite dissatisfied, and neither satisfied nor dissatisfied” if 1–3 points were scored and “Quite satisfied and very satisfied” if 4–5 points were scored.

### The Instrument

The questionnaire was adapted from an internationally validated tool for the assessment of parent experience and satisfaction in the NICUs called the EMPATHIC-N Instrument, which consisted of 57 items (3). Permission to translate and adapt the original questionnaire was obtained from the author. A short version of the instrument was developed, considering the previous experience in reducing a similar questionnaire related to the Pediatric Intensive Care Unit (23). During the reduction process, attention was paid to keep the original balance among the different items for each domain of the questionnaire. The project team working on quality improvement in the three NICUs was involved in this reduction process. Moreover, the medical director of one of the involved hospitals, a NICU pediatrician and President of the Ethiopian Pediatric Society participated in this task.

All the suggested steps for translation and cultural adaptation were followed (24, 25). Starting from the 57 items of the EMPATHIC-N questionnaire (English version), 19 items were considered inappropriate for the Ethiopian setting both for cultural and organizational reasons. The reasons for the removal of the items are reported in Supplementary File 1. Similarly, the original final questions regarding overall satisfaction with physicians and nurses (Q41 and Q42) from the original EMPATHIC-N were substituted with more general questions evaluating care and treatment received from HCPs by the infant and the parent, retrieved from another validated questionnaire on NICU parents' satisfaction, the NSS-13 tool (26). The questionnaire also included four open questions to collect comments or suggestions from the parents about their experience with care received in the NICU during admission, stay, and discharge plus a general comment on the service received.

The instrument was translated into the two languages mainly spoken in the areas where the three hospitals were located—Afan Oromo and Amharic—and back-translated into English to check for consistency. Finally, a pre-test was administered on a small sample (*n* = 10) of the target population to assess the comprehensibility of the instrument questions in the two language versions.

### Data Collection

The study was conducted between July and October 2019. Three health care providers, who had no direct connection with the service, were recruited as data collectors and received a 1-day training session about the study tool and methodology. The principal investigator supervised the data collection processes. The interviewer invited the parents to participate in the survey and to complete the questionnaire upon discharge from the NICU. The interview was conducted in a previously identified quiet location within the hospital and lasted on average of 20–30 min. The interviewer marked the respondent's response on paper questionnaires and literally transcribed the answers given to the open-ended questions. Then the data collector copied the answers into an electronic database. The interviews were not audio recorded. A number code was sequentially assigned to the enrolled parents to ensure anonymity.

### Data Analysis

Statistical analysis was conducted with R software (R version 4.0.3). Data coding, clearance and entry to the designed format was done after completing data collection.

Descriptive analyses exploring participants' characteristics were included for the total sample and separately for the three NICU levels. Frequencies and percentages were reported for categorical variables, and medians with the interquartile range (iqr) for continuous variables. Raw means and standard deviations were included to describe single item results in the sample.

The Kaiser-Meyer-Olkin (KMO) test of factorial adequacy and Bartlett's test were used to measure the factorability of the correlation matrix.

Confirmatory factor analysis (CFA) was conducted to examine the validity of EMPATHIC-N in this study. Five construct domains were identified as latent variables, which had been defined according to correspondent items in the original EMPATHIC-N instrument. Goodness of fit was investigated considering standard measures such as Comparative Fit Index (CFI;), Root Mean Square Error of Approximation (RMSEA), and Standardized Root Mean Square Residual (SRMR) (27). The reliability of each factor was examined through internal consistency using Cronbach's alpha coefficients. Congruent validity was examined by correlating the domains of the questionnaire with the four general questions on parents' overall experience and impression.

Univariate associations between participants' sociodemographic characteristics and mean scores for the five domains and for a total satisfaction score (calculated as overall mean for all item scores), and for the dichotomized general questions on overall experience and impression (Q39–Q42) were investigated. The Wilcoxon rank-sum and Kruskal Wallis tests were used for continuous variables and Fisher's exact tests was used for categorical variables. Multiple median regression and logistic regression models were additionally implemented to model multivariate associations between parents and patients' characteristics (age, residence, education, occupation, length of hospital stay) and the outcomes (domain scores and binary outcomes representing the four general satisfaction items agreement). The level of statistical significance was set at *p* < 0.05.

Qualitative data were collected from the open-ended questions and analyzed through inductive content analysis (28). The contents of the answers were compiled and coded into subthemes independently by the principal investigator and a co-investigator. The researchers created a consolidated checklist that was used to independently apply the coding. The subthemes were categorized into 6 themes: General reaction, Communication, Relationship with Health Workers, Clinical management, Organizational aspects and Environmental factors.

## Results

### Characteristics of the Study Participants

Of the 418 parents who were invited to participate in the study, 386 accepted. Therefore, the overall response rate was 92%. Specifically 91% (*n* = 32 out of 35) in NICU Level I, 94% (*n* = 117 out of 124) in Level II and 92% (*n* = 237 out of 258) in Level III. Most of the questionnaires were filled in Amharic (71.8%, n. 277). Almost all the respondents were the mothers (98.7%, n. 381), most of them were married (97.7%, n. 378) and were housewives (64%, n. 248). Table 1 reports the distribution of participants' socio-demographic characteristics both in the whole sample and across the NICU levels. Only the 9.8% of the responders' infants were hospitalized for more than 2 weeks.

**Table 1:** Socio-demographic characteristics of the respondents and child admission variables (*N* = 386) in the whole sample and across the three NICU levels.

**Variable**	**N**	**Level I**	**Level II**	**Level III**	**Whole sample**	***P^a^***
		**(*n* = 32)**	**(*n* = 117)**	**(*n* = 237)**	**(*n* = 386)**	
**Age, Median (q1, q3)^b^**	386	25.5 (21.5, 30.5)	26.0 (22.0, 30.0)	26.0 (24.0, 29.0)	26.0 (24.0, 29.0)	0.56
**Language**, ***n*** **(%)**	386					<0.001
Amharic		5 (15.6)	35 (29.9)	237 (100.0)	277 (71.8)	
Afan Oromo		27 (84.4)	82 (70.1)	0 (0.0)	109 (28.2)	
**Residence**, ***n*** **(%)**	380					<0.001
Rural		21 (70.0)	70 (61.9)	37 (15.6)	128 (33.7)	
Urban		9 (30.0)	43 (38.1)	200 (84.4)	252 (66.3)	
**Education**, ***n*** **(%)**	386					
No education		9 (0.3)	38 (0.3)	29 (0.1)	76 (19.7)	<0.001
Low education		13 (0.4)	38 (0.3)	83 (0.4)	134 (34.7)	
High education		10 (0.3)	41 (0.4)	125 (0.5)	176 (45.6)	
**Occupation**, ***n*** **(%)**						0.08
Homeworking		22 (68.8)	84 (71.8)	142 (59.9)	248 (64.2)	
Other		10 (31.2)	33 (28.2)	95 (40.1)	138 (35.8)	
**Parent gender**, ***n*** **(%)**	386					0.78
Father		5 (1.3)	2 (1.7)	0 (0.0)	3 (1.3)	
Mother		381 (98.7)	115 (98.3)	32 (100.0)	234 (98.7)	
**Child gender**, ***n*** **(%)**	386					0.41
Male		20 (62.5)	70 (59.8)	127 (53.6)	217 (56.2)	
Female		12 (37.5)	47 (40.2)	110 (46.4)	169 (43.8)	
**Length of stay**, ***n*** **(%)**	386					0.04
<1 week		15 (46.9)	64 (54.7)	152 (64.1)	231 (59.8)	
1–2 weeks		13 (40.6)	45 (38.5)	59 (24.9)	117 (30.3)	
>2 weeks		4 (12.5)	8 (6.8)	26 (11.0)	38 (9.8)	

a *Kruskal test for continuous variables and Fisher tests for categorical variables*.

b *q1 = first percentile, q3 = third percentile*.

Parents' spoken language, place of residence, education level and children's length of stay were found to be differently distributed across the NICU levels (Table 1).

### Instrument Validation

Questions 12 (“We were daily informed about the physicians and the nurses who were in charge of our child”) and 29 (“Nurses and physician always identified themselves saying their name and their role”) were not answered consistently. Low item-total correlations (*r* = 0.02 and *r* = 0.03 respectively) and higher “alpha-drop” estimates (Cronbach's alpha for a group of items if each item were dropped) showed that items were not correlating well with the scale overall.

The Kaiser-Meyer-Olkin (KMO) statistic measure was 0.94 and Bartlett's test resulted significative. Confirmatory factor analysis (CFA) was performed for the whole sample and separately for the two languages.

In line with the original instrument (3) and with the following adapted versions (2, 8, 29, 30), five latent factors were specified (Communication, Care & Treatment, Parental Participation, Organization, Professional Attitude). Since all factors were highly correlated (Supplementary File 2; Supplementary Table 1), the model was re-specified by defining a second order factor measured by the five domains of the questionnaire. Model goodness of fit is reported in Table 2, indices were not optimal but comparable with results from previous studies (29). Second-order CFA Factor loadings are available in the Supplementary File 2; Supplementary Table 2.

**Table 2:** Confirmatory factor analyses results.

**Measures of fit^a^**	**First order CFA**			**Second order CFA**		
	**Amharic**	**Afan Oromo**	**Whole sample**	**Amharic**	**Afan Oromo**	**Whole sample**
CFI	0.81	0.71	0.80	0.80	0.70	0.79
RMSEA	0.06	0.10	0.06	0.07	0.10	0.07
SRMR	0.07	0.10	0.07	0.08	0.15	0.09

a *CFI, comparative fit index; RMSEA, root mean square error of approximation; CFA, confirmatory factor analysis; TLI, Tucker–Lewis index*.

*A good model fit was attained for CFI close to 1, RMSEA <0.06 and SRMR <0.08*.

Cronbach's alpha coefficients for the two language sub-samples and for the whole sample analyses (Afan Oromo and Amharic questionnaires combined) were very high (>0.70), confirming the reliability of all the five construct domains across the three combinations (Table 3).

**Table 3:** Cronbach's alpha internal consistency for each of the five construct domains, separately for the two language sample subsets and for the total sample.

	**Amharic**	**Afan Oromo**	**Total sample**
Communication	0.84	0.89	0.86
(information and education)			
Care and treatment	0.90	0.87	0.90
Parental participation	0.77	0.77	0.74
Organization/hospital environment	0.76	0.92	0.79
Professional attitude	0.88	0.94	0.90
**Overall measure**	0.95	0.96	0.95

There was congruence between the results obtained in the two language samples and in the combined sample. For this reason, the authors decided to describe the questionnaire outputs in the overall sample of respondents, without distinguishing them by language.

Finally, correlations between the five domain scores and the scores of items Q39–Q42, regarding general questions on parents' overall experience and impression, were all significant (Supplementary File 2; Supplementary Table 5).

### Parent Satisfaction and Association With the NICU Level

For almost all the items, parent satisfaction with the quality of care received in the NICU, the mean scores were higher than four on the 6-point Likert scale (Supplementary File 2; Supplementary Table 3).

The parents of children in the Level II NICU consistently reported higher mean scores in all the questionnaire items and for all the five domains. In the analysis of the five item-aggregated latent domains (Q12 and Q29 excluded) the highest mean scores were obtained (in descendent order) for Parental participation [median 5.17; iqr (4.67, 5.62)], Professional Attitude [median 5.11; iqr (4.67, 5.67)] and Care and Treatment [median 5.00; iqr (4.62, 5.50)]; while the total scores were relatively lower for the domain areas of Communication [median 4.71; iqr (4.00, 5.14)] and Organization/Hospital environment [median 4.67; iqr (4.33, 5.17)] (Table 4).

**Table 4:** Domains Scores and total satisfaction score per each site (*N* = 386).

**Variable, median (q1, q3)**	**Level I**	**Level II**	**Level III**	**Overall sample**	***P^a^***
	**(*n* = 32)**	**(*n* = 117)**	**(*n* = 237)**	**(*n* = 386)**	
Communication (Information and education)	3.79 (2.68, 4.64)	5.29 (4.86, 5.71)	4.57 (3.86, 4.86)	4.71 (4.00, 5.14)	* <0.001*
Care and treatment	4.75 (4.38, 5.03)	5.62 (5.25, 6.00)	4.88 (4.38, 5.00)	5.00 (4.62, 5.50)	* <0.001*
Parental participation	4.37 (3.96, 4.83)	5.17 (5.00, 6.00)	5.17 (4.83, 5.50)	5.17 (4.67, 5.62)	* <0.001*
Organization/ hospital environment	4.50 (4.33, 4.83)	6.00 (5.00, 6.00)	4.50 (4.17, 4.67)	4.67 (4.33, 5.17)	* <0.001*
Professional attitude	4.00 (3.64, 4.25)	5.89 (5.44, 6.00)	5.00 (4.67, 5.22)	5.11 (4.67, 5.67)	* <0.001*
Total satisfaction score ^b^	4.25 (3.96, 4.50)	5.56 (5.14, 5.81)	4.83 (4.44, 5.03)	4.92 (4.53, 5.38)	* <0.001*

a *Kruskal Wallis tests*.

b *Calculated as mean of all the items scores*.

Table 5 shows statistic of the domain scores according to the participants' characteristics. The total satisfaction score varied according to participants' language, place of residence, and level of education. Multivariate analyses showed consistent results with univariate analyses (Supplementary File 2; Supplementary Table 6). The language variable was not considered in the models because Afan Oromo was not spoken in one of the three hospitals (Table 1). Satisfaction scores were always reported to be significantly lower in Level I and Level III NICUs compared to Level II NICU (reference category).

**Table 5:** Statistics of the domain scores according to participants' characteristics.

**Sample characteristic, median (q1, q3)**	**Communication**	**Care and Treatment**	**Parental Participation**	**Organization/Hospital environment**	**Professional Attitude**	**Total satisfaction score**
**Age**
≤ 26 years	4.71 (4.00, 5.07)	5.00 (4.62, 5.56)	5.17 (4.67, 5.50)	4.83 (4.33, 5.17)	5.11 (4.78, 5.67)	4.97 (4.58, 5.38)
>26 years	4.71 (4.00, 5.14)	4.88 (4.62, 5.50)	5.17 (4.67, 5.67)	4.50 (4.17, 5.17)	5.11 (4.56, 5.67)	4.89 (4.47, 5.38)
*P^a^*	*0.63*	*0.15*	*0.27*	*0.02*	*0.48*	*0.36*
**Language**
Amharic	4.57 (3.86, 4.86)	4.88 (4.50, 5.25)	5.17 (4.83, 5.50)	4.50 (4.17, 5.00)	5.11 (4.67, 5.22)	4.86 (4.47, 5.11)
Afan Oromo	5.00 (4.57, 5.57)	5.50 (5.00, 5.88)	5.00 (4.40, 6.00)	5.17 (5.00, 6.00)	5.56 (4.67, 6.00)	5.22 (4.61, 5.78)
*P^a^*	* <0.001*	* <0.001*	*0.56*	* <0.001*	* <0.001*	* <0.001*
**Residence**
Rural	5.00 (4.39, 5.43)	5.19 (4.75, 5.75)	5.17 (4.50, 5.71)	5.00 (4.50, 6.00)	5.22 (4.86, 6.00)	5.11 (4.69, 5.64)
Urban	4.57 (3.96, 4.86)	4.88 (4.50, 5.25)	5.17 (4.67, 5.50)	4.67 (4.17, 5.00)	5.00 (4.67, 5.25)	4.86 (4.47, 5.14)
*P^a^*	* <0.001*	* <0.001*	*0.39*	* <0.001*	* <0.001*	* <0.001*
**Education**
No education	4.86 (4.25, 5.43)	5.00 (4.84, 5.62)	5.00 (4.67, 5.38)	5.00 (4.46, 5.33)	5.11 (4.97, 5.78)	4.96 (4.58, 5.47)
Low education	4.71 (4.00, 5.14)	5.00 (4.75, 5.62)	5.33 (4.88, 5.67)	4.67 (4.33, 5.46)	5.11 (4.81, 5.78)	4.92 (4.62, 5.52)
High education	4.64 (3.86, 5.00)	4.88 (4.38, 5.50)	5.00 (4.50, 5.50)	4.67 (4.17, 5.00)	5.11 (4.44, 5.36)	4.89 (4.38, 5.18)
*P^b^*	*0.15*	*0.004*	*0.009*	*0.03*	*0.04*	*0.06*
**Occupation**
Housewife	4.71 (4.00, 5.14)	5.00 (4.72, 5.50)	5.17 (4.83, 5.54)	4.67 (4.33, 5.17)	5.11 (4.78, 5.67)	4.92 (4.58, 5.42)
Other	4.57 (4.00, 5.00)	5.00 (4.38, 5.62)	5.17 (4.50, 5.62)	4.67 (4.17, 5.00)	5.11 (4.44, 5.56)	4.89 (4.33, 5.30)
*P^a^*	*0.12*	*0.15*	*0.3*	*0.06*	*0.13*	*0.13*
**Length of stay**
<1 week	4.71 (4.00, 5.14)	5.00 (4.62, 5.62)	5.17 (4.67, 5.67)	4.67 (4.33, 5.17)	5.11 (4.78, 5.67)	4.92 (4.56, 5.35)
1–2 weeks	4.71 (4.14, 5.14)	5.00 (4.62, 5.62)	5.17 (4.50, 5.50)	4.83 (4.33, 5.83)	5.11 (4.78, 5.78)	4.92 (4.53, 5.47)
>2 weeks	4.64 (3.86, 4.86)	4.69 (4.25, 5.00)	5.17 (4.71, 5.67)	4.50 (3.88, 4.83)	5.00 (4.33, 5.22)	4.81 (4.17, 5.06)
*P^b^*	*0.59*	*0.01*	*0.39*	*0.11*	*0.11*	*0.24*

a *Wilcoxon rank sum*.

b *Kruskal tests*.

Table 6 reports frequencies and percentages for the four general questions, concerning parents' overall experience and impression. For the two “general experience” questions and the two “general impression” questions, the percentage of parents satisfied with the level of care in Level II NICU was higher than 90%. In the Level II NICU, for questions 39 and 40 almost 100% of the parents were very or totally satisfied, and for questions 41 and 42, 95 and 90%, respectively, of the parents were quite satisfied or very satisfied. Descriptions of the overall experience and impression scores in the original undichotomized scales are reported in Supplementary File 2; Supplementary Table 4.

**Table 6:** General questions on parents' experience and impression.

**Variable, *n* (%)**	**Level I**	**Level II**	**Level III**	**Overall sample**	***P^a^***
	**(*n* = 32)**	**(*n* = 117)**	**(*n* = 237)**	**(*n* = 386)**	
**Overall experience**					
1. We would recommend this NICU to anyone facing a similar situation					* <0.001*
Not at all/to a small extent/to some extent/to a large extent	12 (37.5)	2 (1.7)	47 (19.8)	61 (15.8)	
To a very large extent/all the time	20 (62.5)	115 (98.3)	190 (80.2)	325 (84.2)	
2. If ever we would get in the same situation again, we would like to come back to this NICU					* <0.001*
Not at all/to a small extent/to some extent/to a large extent	8 (25.0)	2 (1.7)	46 (19.4)	56 (14.5)	
To a very large extent/all the time	24 (75.0)	115 (98.3)	191 (80.6)	330 (5.5)	
**Overall impression**
3. All in all, how satisfied or dissatisfied are you with the treatment the child received at the NICU?					* <0.001*
Very dissatisfied /dissatisfied /quite dissatisfied/neither satisfied nor dissatisfied	5 (15.6)	6 (5.1)	45 (19.0)	56 (14.5)	
Quite satisfied/very satisfied	27 (84.4)	111 (94.9)	192 (81.0)	330 (85.5)	
4. All in all, how satisfied or dissatisfied are you with how you were treated as a parent?					* <0.001*
Very dissatisfied /dissatisfied /quiet dissatisfied/neither satisfied nor dissatisfied	12 (37.5)	11 (9.4)	46 (19.4)	69 (17.9)	
Quite satisfied/very satisfied	20 (62.5)	106 (90.6)	191 (80.6)	317 (82.1)	

a *Fisher exact tests*.

NICU level, level of education and occupation were found to be possibly associated to a different resulting outcome in general question outcomes (Supplementary File 2; Supplementary Table 7). Additionally, language and place of residence were found to be possibly associated with answers to question Q40. Multivariate logistic regression analyses (Supplementary File 2; Supplementary Table 8) confirmed, for the four outcomes, that the odds of being very largely/all the time in agreement with Q39 and Q40 and quite/largely satisfied with Q41 and Q42 were lower in Level I and III NICUs compared to the Level II NICU, while holding the other variables constant.

### Open Questions About Parents' Experience

All the participants answered to at least three of the four open-ended questions, providing 1,541 answers. Fourteen comments contained two different subthemes generating a total of 1,555 comments.

Aspects related to admission, stay and discharge were often reported in the “General experience” question, therefore the authors decided to code together all the comments given to the four questions.

Most of the comments were simple opinions or statements about personal feelings. The main aspects commented in relation to the experience were: information received (227 respondents), speed of treatment (207 respondents), and HCPs' behavior (110 respondents). The complete list of the subthemes is reported in Table 7.

**Table 7:** Themes and selected verbatim responses to open questions about parents' experience.

**Theme**	**Sub theme**	**Participants**	**Verbatim data extracts (Positive and Negative comments)**
General reaction	General opinion	378	*Pos (360): “It was nice/great/perfect/wonderful/good/not bad” “No problem”* *Neg (10): “I can't say that it was good” “Not good. But I will come here If any problem because I have no choice”*
	Feeling	205	*Pos (205): “I'm happy/glad” “Leave with happiness”*
	Thanks	129	*Pos (129): “Thank you” “May god bless all of them”*
	Recommendations	67	*Pos (66): “Keep up the good work”, “Let the service continue as it is”* *Neg (1): “I wish they could do more to improve”*
	Description	24	*Pos (24): “I delivered in this hospital” “They took my baby with care and provided the service”*
Communication	Language	3	*Neg (3): “I have a language problem. Some of them don't understand me”*
	Information received	227	*Pos (205): “They listen to me and answer my question properly”,“I got a good response for my questions”, “I got health education on how to care for my baby”* *Neg (22): “When I asked about my baby's condition some nurses gave me the wrong information”, “They didn't discuss with the mother”, “No needful advice”*
Relationship with health workers	Behavioral	110	*Pos (91): “They were welcoming” “They treated me well/with love and care”* *Neg (19): “They didn't have any sympathy” “Some nurses have no respect for mothers”*
Clinical management	Care	56	*Pos (38): “My baby got good care and treatment” “Compared to the health center, the care and treatment is very good”, “Preparation service is good”* *Neg (18): “I don't believe my baby got good care”, “Some nurses did not follow children well especially during night time”, “There is negligence in the night shift”*
	Equality	8	*Neg (8): “The service should be the same for everyone” “I Suggest If we get equal care and treatment from all the nurses all the time”*
Organizational aspects	Speed of treatment	207	*Pos (203): “My child was admitted soon” “They were fast”* *Neg (4): “There was a problem during admission, I was waiting”, “Not fast”*
	Services	20	*Neg (20): “There is a problem in the laboratory, they were taking our blood outside the hospital” “There is no good coordination to get laboratory results”, “Even the oxygen is outside”*
	Equity	3	*Pos (3): “We got help for free it is good especially for poor people”*
	Priority	1	*Neg (1): “I wish the ward room service gave priority to infant patients”*
Environmental factors	Comfort	96	*Pos (87): “The place was comfortable” “I felt comfortable”* *Neg (9): “It doesn't give me comfort”*
	Equipment	17	*Neg (17): “There are no beds for mothers, especially for mothers who delivered”, “I slept on the chair/on the floor”, “There are not enough gowns and caps”*
	Space	3	*Neg (3): “I wish there were additional rooms” “The room is too small”*
	Cleanness	1	*Neg (1): “The Kangaroo Mother Care room is not clean”*

## Discussion

This study aimed to investigate parents' experience and satisfaction with the care received in three different level NICUs in Ethiopia. Therefore, two language translations, cultural adaptation and validation of the questionnaire EMPATHIC-N (3) were performed in this setting.

The lack of attention of parents in distinguishing the health professional figures who were responsible for the care of the newborn had already been taken into account during the adaptation of the questionnaire, but was further confirmed by the non-correlation of questions 12 and 29 relating to the identification of health personnel. This study had a higher response rate (94%) compared to other studies (2, 23), similarly to one study conducted in a Pediatric ICU in Spain where the questionnaire was administered at the time of discharge (30). This high response rate could be partly explained by the data collection method (direct interviews instead of self-compilation) and by the level of satisfaction across all the sites, confirming that people who report high satisfaction levels are more likely to participate in a survey (31).

Most of the respondents were mothers. Such a high percentage of mothers compared to fathers was also revealed in the validation of EMPATHIC in Turkey (29) where—unlike other contexts where both parents were given the opportunity to respond together (2, 10, 11)—the couple was free to choose whether the mother or the father wished to answer. This may also because mothers are more present in hospitals and more involved in the care of newborns, especially in low-resource settings.

In the whole sample, parents resulted to be altogether satisfied with the care received in NICUs. Questionnaire items scored similarly to those of previous studies conducted in different international contexts with average scores even higher than the averages we found (2, 11, 30). These results may suggest a possible ceiling effect that could be due to the parents' feelings of gratitude at the time of their infant's discharge. The implementation of a different scale could be tested in future studies for EMPATHIC-N.

In this study setting, parental participation scored highest among all the five questionnaire domains, whereas the organization/hospital environment domain received the lowest scores for parent satisfaction. This was not highlighted in other study settings (2, 8, 29, 30, 32).

The scores across the five domains were found to vary significantly across the three NICU levels (Level I, II, and III).

Parents of infants assisted in the Level II NICU, expressed a higher level of satisfaction, in all domains, after adjusting the results for parents' socio-demographic characteristics. Instead, satisfaction with Level I NICU resulted in scores lower than four, especially for Communication and Professional Attitude domains. This evidence might be due to the different NICU levels, to whether the hospitals were public or private and to the intervention in the level II NICU supported by CUAMM.

From the point of view of the NICU level it can be assumed that the intermediate level was the one that satisfied parents most, because it offered more services than level I, but did not suffer from heavy workload like in level III (33).

In the literature it has been shown how drugs and medical equipment availability (13), availability of laboratory, radiology services and rooms for accommodation (19) can influence patients' satisfaction. On the other hand, there is evidence that parental satisfaction in provincial centers is higher than in national specialist centers, probably due to the lower severity of cases and less stressful working conditions (34, 35). Moreover, a greater satisfaction of patients in hospitals with a private-public-partnership than in public hospitals has already been previously reported (36). Among other aspects this may have influenced parental satisfaction with discharge preparation (37) and health workers' care and behavior (38, 39). In fact, there is also evidence that the working environment and infrastructure could motivate HCPs to deliver high quality of care (40).

The respondents' level of education was found to be inversely associated with the level of satisfaction, as already reported by other authors (12, 41). This might indicate that parents with a higher level of education have a greater expectations regarding the quality of care provided to their newborn.

Similarly, it has been shown that place of residence might influence satisfaction, whereby people living in rural areas tend to be more satisfied compared to those living in urban areas (12, 41). Levels of parent satisfaction in Care and Treatment for their children were also found to be lower for longer lengths of hospital stay. As also reported by other authors, longer hospitalization tends to be associated to lower satisfaction ratings (42). This could also be due to the greater seriousness of the clinical conditions of the infants who stayed longer in the NICU, and parents had more time to observe the NICU environment and its weaknesses. Therefore, the evaluations by parents who were older, more educated, lived in urban areas, and whose child had a longer length of stay in the NICU, should be used as benchmarks to guide the improvement interventions. In fact, the needs of this population should constitute the basis for more targeted health services for infants and parents in NICUs also in Ethiopia.

In the open questions, parents often gave either positive or negative opinions, but there were many who pointed out possible areas for improvement, even without expressing a direct judgment.

Much attention was paid to the relationships they built with the hospital staff (information received and HCPs' behavior) as also highlighted in other contexts (11).

Positive comments can be very useful to share with the staff to reinforce trust in the HCPs. On the other hand, criticism can be useful to policy makers to improve and make specific interventions. These findings could be used to prompt further discussion, consideration and evaluation for themes such as the cultural approach methodology to apply in these settings. The staff would then be invited to evaluate the parents' feedback, understand any issues and gaps they might encounter and find solutions to address them.

Comments to open questions have also been made available in disaggregate versions (for each study site) to stakeholders and policy makers, but were not presented in this paper.

## Limitations

This survey was conducted upon discharge of the patient from the NICU. Although investigators tried to conduct the interview only after discharge was completed, guaranteeing the anonymity of the interview, being still in the hospital setting may have induced fear of retaliation from the medical and nursing staff for negative or non-favorable reports on the care they provided (11).

The study design provided for a long period (45–60 days) of data collection. This could have led the data collector to develop emotional attachment with the NICU staff and therefore could have prompted staff to improve in their actions and impressions with the parents. To minimize it, data collectors were asked to limit their interactions with staff.

Despite the attempt to guarantee the privacy of the parents during the data collection interview, it could be possible that noise and distractions in the NICU may have led to environmental bias, not allowing the parent to fully concentrate on the questions of the survey. Moreover, collecting data in the same day the infants were discharged could have overwhelmed the parents, but this was the same timing used in similar studies (2).

The data collection tool used in this study, the 6 point-likert scale questionnaire, was not originally designed to be administered by interviewer (3, 23), but as a self-administered questionnaire. The approach/questioning of the interviewer to make clarification on the scales combined with the low level of educational status of the respondents may have led to response bias. To minimize this issue, the interviewers were oriented prior to data collection to suggest respondents to choose between numbers 1 to 6 indicating that 1 for “*Not at all”* to 6 for “*All the time”*.

When comparing satisfaction across the different NICU levels, the health personnel staffing levels and their job satisfaction, which may have affected their care activities, were not taken into consideration. Future studies on this issue will also need to take these aspects into account.

The role played by public or private hospital ownership in the provision of care was not been investigated in the present study. Further insights are needed to distinguish whether private non-profit NICU facilities led to better parent satisfaction than the public ones investigated in this paper.

In addition, some of the parents' characteristics that may have influenced their experience were not considered in our study, such as having other children at home to care for or having had previous experience with NICU services.

## Conclusion

In the present study, we found that the EMPATHIC-N questionnaire can be applied to measure parental satisfaction and can be adapted to different cultural contexts. This study investigated parents' satisfaction in Ethiopia, also comparing results across three different NICU levels. Parents' overall level of satisfaction with the care provided to their child was good, in particular with regard to “Parental participation.” Another interesting finding of this study was that healthcare workers tended not to give much importance to introducing themselves to the patients and their parents. In addition, we found that different domains in the healthcare service might influence the level of parent satisfaction according to characteristics such as education and place of residence. Healthcare team members need to be aware of the increasing importance of identifying parental expectations and understanding its significance. In addition to the clinical care they provide to the newborn, the healthcare staff must also consider the needs of parents as part of their daily practice.

Further studies are needed to improve staff awareness about parent satisfaction and more precisely on their evaluation of aspects related to the care provided across the different NICU levels. Long-term investigation is also recommended to better evaluate and understand differences in the way care is provided across the different NICU levels in Ethiopia.

## Data Availability Statement

The raw data supporting the conclusions of this article will be made available by the authors, without undue reservation.

## Ethics Statement

The studies involving human participants were reviewed and approved by St. Paul's Hospital Millennium Medical College/Oromia Regional Health Bureau. Verbal informed consent was obtained from all participants for their participation in this study.

## Author Contributions

BG and LM conceived and implemented the study, collected data, participated in the data analysis, and drafted the manuscript. FT drafted and edited the manuscript and participated in the interpretation of the findings. SP performed the data analysis, gave a substantial contribution to the interpretation of the data, and drafted and edited the manuscript. AT and FM supervised all the phases of study and made a substantial contribution to the interpretation of the data. TA reviewed and gave substantial contribution to the manuscript. ID conceived, designed and supervised all the phases of study, and drafted and revised the manuscript for important intellectual content. All authors contributed, read, and approved the final manuscript.

## Funding

This research was conducted in the framework of project supported by Italian Agency for Development Cooperation (AICS) (Grant No. AID 11512/CUAMM/ETH). The project supported the research costs and the publication fees. The founders had no role in study design, data collection, data analysis, data interpretation, or writing of the report.

## Conflict of Interest

The authors declare that the research was conducted in the absence of any commercial or financial relationships that could be construed as a potential conflict of interest. The Handling Editor declared a past collaboration with one of the authors, ID.

## Publisher's Note

All claims expressed in this article are solely those of the authors and do not necessarily represent those of their affiliated organizations, or those of the publisher, the editors and the reviewers. Any product that may be evaluated in this article, or claim that may be made by its manufacturer, is not guaranteed or endorsed by the publisher.
