# Supplementary material for: Parents' Experience and Satisfaction in Neonatal Intensive Care Units in Ethiopia: A Multicenter Cross-Sectional Study Using an Adapted Version of EMPATHIC-N
Source: Front Pediatr. 2021 Oct 8;9:738863. doi: 10.3389/fped.2021.738863 (PMC8531748; doi:10.3389/fped.2021.738863)
Supplement: Supplementary file 1 [file Data_Sheet_1.pdf]

Supplementary Materials

Supplementary file 1: EMPATHIC-N excluded items and reasons for their removal.

	EMPATHIC-N questions (57 items)	Reasons for removal	Comments
	**Information**		
3	The information given by the doctors and nurses was always the same	Cultural	Parents often do not distinguish the different types of professionals; they did not know who were the nurses and who the doctors.
7	The doctors and nurses gave understandable information about the examinations and tests	Cultural	Explanations given to parents usually did provide details about the tests, but focused on the health conditions of the newborn
8	We received understandable information about the effects of the drugs by the doctors and nurses	Cultural	Explanations given to parents were usually not detailed. Question 5 "*The doctor clearly informed us about the consequences of our child's treatment*" was preferred because it specifically refers to the child's health
10	The information brochure we received was complete and clear	Organizational	No brochures are available in hospitals in Ethiopia to inform parents
12	The doctors and nurses gave honest information to us	Cultural	Parents do not feel they are in the position to judge doctors' / Nurses' truthfulness
	**Care & Treatment**		
14	The team was alert to the prevention and treatment of pain in our child	Organizational	Since parents are not allowed to stay constantly inside the NICU, they do not have the possibility to understand the specific meaning of pain treatment and whether it was timely for their child
15	The doctors and nurses are real professionals; they know what they are doing	Cultural	Parents do not feel they are in the position to judge doctors' / nurses' preparation and competencies.
16	The correct medication was always given on time	Organizational	Since parents are not allowed to stay constantly inside the NICU, they do not have the possibility realize whether the treatment given to their child is correct and timely.
17	At admission, our child's medical history was known by the doctors and nurses	Cultural	Parents do not feel they are in the position to judge doctors' / nurses' preparation and competencies.
18	Attention was paid to our child's developmental by the doctors and nurses	Cultural	Parents are not aware of the developmental effects of childhood illness

21	The team had a common goal: the best care and treatment for our child and ourselves	Cultural	Parents do not feel they are in the position to judge doctors' / nurses' commitment
26	The team was caring to our child and to us	Cultural	Very general question, probably all parents would have answered positively. We preferred to keep more specific questions about emotional support (question 13) or needs (question 14).
29	The transfer of care from the NICU staff to colleagues in the pediatric ward had gone well	Organizational	There is usually no transfer from the NICU to the pediatric ward
	**Parental participation**		
33	Even during intensive procedures we could always stay close to our child	Organizational	Parents are not allowed to stay constantly inside the NICU
35	The nurses helped us in the bonding with our child	Organizational	Parents are not allowed to stay constantly inside the NICU
	**Organization**		
41	The unit could easily be reached by telephone	Organizational	Parents have no access to telephone services.
44	Noise in the unit was muffled as good as possible	Cultural	Noise was not considered to have an impact on their comfort
	**Professional Attitude**		
51	At our bedside, the discussion between the doctors and nurses was only about our child	Organizational	Since parents are not allowed to stay constantly inside the NICU, they could not listen to discussions between doctors and nurses.
56	Our child's health always came first for the doctors and nurses	Cultural	Very general question, probably all parents would have answered positively.
