# Supplementary material for: Parents' Experience and Satisfaction in Neonatal Intensive Care Units in Ethiopia: A Multicenter Cross-Sectional Study Using an Adapted Version of EMPATHIC-N
Source: Front Pediatr. 2021 Oct 8;9:738863. doi: 10.3389/fped.2021.738863 (PMC8531748; doi:10.3389/fped.2021.738863)
Supplement: Supplementary file 2 [file Data_Sheet_2.pdf]

Supplementary Materials

Supplementary file 2

Supplementary Table 1. Pearson's correlations among domains for the whole sample of responders (N=386)

	**Communication**	**Care and Treatment**	**Parental Participation**	**Organization/ Hospital environment**	**Professional Attitude**
**Communication**	1				
**Care and Treatment**	0.82	1			
**Parental Participation**	0.87	0.91	1		
**Organization/Hospital environment**	0.71	0.88	0.85	1	
**Professional Attitude**	0.81	0.91	0.89	0.84	1

*all correlations were statistically significant; p<0.001

Supplementary Table 2 CFA factor loadings

	**Whole Sample**					**Amharic**					**Afam Oromo**				
	**Communication**	**Care and Treatment**	**Parental Participation**	**Organization/ Hospital environment**	**Professional Attitude**	**Communication**	**Care and Treatment**	**Parental Participation**	**Organization/ Hospital environment**	**Professional Attitude**	**Communication**	**Care and Treatment**	**Parental Participation**	**Organization/ Hospital environment**	**Professional Attitude**
**Q1**	0.50	0	0	0	0	0.45	0	0	0	0	0.60	0	0	0	0
**Q2**	0.80	0	0	0	0	0.81	0	0	0	0	0.79	0	0	0	0
**Q3**	0.64	0	0	0	0	0.55	0	0	0	0	0.86	0	0	0	0
**Q4**	0.65	0	0	0	0	0.54	0	0	0	0	0.88	0	0	0	0
**Q5**	0.66	0	0	0	0	0.62	0	0	0	0	0.69	0	0	0	0
**Q6**	0.63	0	0	0	0	0.64	0	0	0	0	0.63	0	0	0	0
**Q7**	0.79	0	0	0	0	0.82	0	0	0	0	0.70	0	0	0	0
**Q8**	0	0.76	0	0	0	0	0.71	0	0	0	0	0.76	0	0	0
**Q9**	0	0.67	0	0	0	0	0.64	0	0	0	0	0.59	0	0	0
**Q10**	0	0.75	0	0	0	0	0.69	0	0	0	0	0.79	0	0	0
**Q11**	0	0.79	0	0	0	0	0.81	0	0	0	0	0.63	0	0	0

**Q13**	0	0.79	0	0	0	0	0.81	0	0	0	0	0.63	0	0	0
**Q14**	0	0.84	0	0	0	0	0.86	0	0	0	0	0.66	0	0	0
**Q15**	0	0.77	0	0	0	0	0.75	0	0	0	0	0.72	0	0	0
**Q16**	0	0.65	0	0	0	0	0.66	0	0	0	0	0.66	0	0	0
**Q17**	0	0	0.53	0	0	0	0	0.48	0	0	0	0	0.84	0	0
**Q18**	0	0	0.21	0	0	0	0	0.24	0	0	0	0	0.79	0	0
**Q19**	0	0	0.40	0	0	0	0	0.37	0	0	0	0	0.86	0	0
**Q20**	0	0	0.81	0	0	0	0	0.83	0	0	0	0	0.46	0	0
**Q21**	0	0	0.55	0	0	0	0	0.66	0	0	0	0	0.51	0	0
**Q22**	0	0	0.32	0	0	0	0	0.51	0	0	0	0	0.52	0	0
**Q23**	0	0	0	0.77	0	0	0	0	0.80	0	0	0	0	0.72	0
**Q24**	0	0	0	0.74	0	0	0	0	0.72	0	0	0	0	0.91	0
**Q25**	0	0	0	0.78	0	0	0	0	0.80	0	0	0	0	0.67	0
**Q26**	0	0	0	0.57	0	0	0	0	0.49	0	0	0	0	0.86	0
**Q27**	0	0	0	0.54	0	0	0	0	0.42	0	0	0	0	0.89	0
**Q28**	0	0	0	0.77	0	0	0	0	0.73	0	0	0	0	0.83	0
**Q30**	0	0	0	0	0.64	0	0	0	0	0.56	0	0	0	0	0.84
**Q31**	0	0	0	0	0.61	0	0	0	0	0.49	0	0	0	0	0.86
**Q32**	0	0	0	0	0.37	0	0	0	0	0.31	0	0	0	0	0.81
**Q33**	0	0	0	0	0.87	0	0	0	0	0.86	0	0	0	0	0.87
**Q34**	0	0	0	0	0.68	0	0	0	0	0.63	0	0	0	0	0.80
**Q35**	0	0	0	0	0.76	0	0	0	0	0.77	0	0	0	0	0.62

**Q36**	0	0	0	0	0.88	0	0	0	0	0.89	0	0	0	0	0.79
**Q37**	0	0	0	0	0.46	0	0	0	0	0.45	0	0	0	0	0.70
**Q38**	0	0	0	0	0.85	0	0	0	0	0.83	0	0	0	0	0.85

Supplementary Table 3. Mean score of the 38 EMPATHIC-N questions (N=38).

**Variable, mean (sd)**	**N**	*Level I (n=32)*	*Level II (n=117)*	*Level III (n=237)*	*Total sample (n=386)*
**Communication (Information and Education)**					
1. We had a daily meeting with the health workers about the medication and the care of our child	386	3.31 (2.04)	5.30 (1.10)	3.52 (1.40)	4.04 (1.61)
2. Health workers answered our questions clearly	386	4.22 (1.43)	5.31 (0.92)	4.35 (1.20)	4.63 (1.23)
3. We were immediately informed in case of worsening of our child's health condition	386	3.75 (1.81)	5.13 (1.00)	4.45 (1.33)	4.60 (1.35)
4. Health workers provided us clear information about our child illness	386	3.31 (1.84)	5.15 (1.07)	4.28 (1.41)	4.46 (1.45)
5. Health workers clearly informed us about the consequences of the treatment administered to our child	386	3.91 (1.57)	4.99 (1.21)	4.26 (1.39)	4.45 (1.40)
6. Health workers informed us about the prospects for our child's future health	386	3.19 (1.97)	4.96 (1.40)	4.55 (1.25)	4.56 (1.44)
7. The information health workers gave us was very clear	386	3.97 (1.47)	5.41 (0.91)	4.61 (1.10)	4.80 (1.17)
**Care and Treatment**					
8. Physician-nurse collaboration was good	386	4.97 (0.74)	5.65 (0.58)	5.11 (0.75)	5.26 (0.75)
9. Health workers took prompt actions in case of worsening of our child's clinical condition	386	4.53 (1.19)	5.59 (0.57)	4.84 (0.92)	5.04 (0.93)
10. Our child's needs were efficiently met	386	4.31 (1.03)	5.51 (0.74)	4.27 (1.11)	4.65 (1.15)
11. Health workers cared about our child's well-being	386	4.56 (1.16)	5.46 (0.65)	4.61 (1.06)	4.87 (1.04)
12. We were informed on a day-to-day basis about the physicians and the nurses who were in charge of our child	386	1.00 (0.00)	1.12 (0.59)	1.64 (1.08)	1.43 (0.94)
13. Health workers emotionally supported us in an appropriate manner	386	4.88 (0.98)	5.57 (0.65)	4.23 (1.35)	4.69 (1.30)
14. Health workers met well our needs	386	4.84 (1.14)	5.62 (0.70)	4.45 (1.16)	4.84 (1.16)
15. A nurse always supported us in case of emergency	386	4.66 (1.26)	5.64 (0.62)	4.81 (0.98)	5.05 (1.00)

16. Nurses always cared about our child cleanness/hygiene and that he/she was comfortable in the incubator	386	4.75 (0.95)	5.50 (0.82)	5.11 (0.84)	5.20 (0.87)
**Parental Participation**					
17. We were actively involved in the decision about our child's care and treatment	386	4.78 (1.34)	5.18 (1.10)	4.16 (1.25)	4.52 (1.30)
18. We were encouraged to stay next to our child	386	4.69 (1.06)	5.32 (1.05)	5.83 (0.53)	5.58 (0.85)
19. Nurses encouraged us to help them while caring for our child.	384	4.58 (0.99)	5.25 (1.05)	5.19 (0.84)	5.16 (0.93)
20. We have confidence in the health staff	386	4.41 (1.34)	5.64 (0.58)	4.49 (1.09)	4.83 (1.12)
21. Nurses taught us how to take care of our newborn child	386	4.31 (1.42)	5.26 (1.15)	5.35 (1.07)	5.23 (1.16)
22. We received information about our child's care before his/her transfer or discharge	386	3.38 (1.66)	4.56 (1.87)	5.40 (1.04)	4.97 (1.52)
**Organization/Hospital environment**					
23. Ward/section of NICU stay gave us a safety sensation	386	4.28 (1.20)	5.56 (0.85)	4.95 (0.66)	5.08 (0.85)
24. Our child incubator or the crib was clean	386	4.66 (0.75)	5.62 (0.55)	5.17 (0.75)	5.26 (0.75)
25. The healthcare staff worked efficiently	386	5.19 (0.74)	5.59 (0.60)	4.79 (0.97)	5.06 (0.93)
26. The space around the incubator/crib was enough	385	4.34 (1.00)	5.51 (0.77)	1.97 (1.07)	3.24 (1.90)
27. The ward/NICU section was clean	386	4.50 (0.88)	5.58 (0.63)	4.94 (1.04)	5.10 (0.98)
28. The atmosphere in the ward/NICU was cordial and without hostilities	386	4.19 (1.09)	5.58 (0.67)	4.73 (0.89)	4.94 (0.96)
**Professional Attitude**					
29. Nurses and physician always introduced themselves by saying their name and their role	386	1.00 (0.00)	1.02 (0.13)	1.14 (0.66)	1.09 (0.53)
30. We received sympathy from health workers	386	3.97 (1.15)	5.64 (0.75)	4.54 (1.11)	4.83 (1.16)
31. The healthcare staff following the hygiene rules	386	4.38 (1.04)	5.62 (0.67)	4.97 (0.79)	5.12 (0.86)
32. The healthcare staff cared about our child and our privacy	386	4.22 (1.26)	5.63 (0.62)	5.77 (0.43)	5.60 (0.73)
33. The healthcare staff showed respect towards our child and us	386	4.28 (1.20)	5.67 (0.59)	4.38 (1.26)	4.76 (1.24)
34. The atmosphere among staff was pleasant	386	4.44 (0.84)	5.72 (0.65)	5.08 (0.63)	5.22 (0.75)
35. We were warmly welcomed by the staff	386	4.56 (0.62)	5.59 (0.74)	4.72 (0.97)	4.97 (0.97)
36. The healthcare staff gave the proper attention to our child and us despite their workload	386	4.44 (0.80)	5.61 (0.64)	4.70 (1.17)	4.96 (1.10)
37. Our culture of origin was taken into account.	386	1.12 (0.55)	5.68 (0.54)	5.02 (0.56)	4.90 (1.30)
38. Health workers were always willing to listen to us	386	3.84 (1.19)	5.61 (0.73)	4.41 (1.31)	4.73 (1.30)

Supplementary Table 4. Overall experience and impression items mean scores.

**Variable, mean (sd)**	**N**	***Level I***	***Level II***	***Level III***	***Overall sample***
		(n=32)	*(n=117)*	*(n=237)*	*(n=386)*
**Overall experience[a] (6-points scale)**					
39. We would recommend this NICU to anyone facing a similar situation	386	4.66 (0.94)	5.21 (0.45)	5.08 (1.31)	5.08 (1.10)
40.If ever we would get in the same situation again, we would like to come back to this NICU	386	4.88 (0.61)	5.25 (0.47)	5.08 (1.33)	5.12 (1.09)
**Overal impression[b] (5-points scale)**					
41.All in all, how satisfied or dissatisfied are you with the treatment the child received at the NICU?	386	3.88 (0.91)	4.66 (0.60)	4.46 (1.01)	4.47 (0.91)
42. All in all, how satisfied or dissatisfied are you with how you weretreated as a parent?	386	3.59 (0.98)	4.60 (0.68)	4.45 (1.01)	4.42 (0.95)

Supplementary Table 5. Pearson's correlations between construct questionnaire domains and overall questions.

	**Communication**	**Care and Treatment**	**Parental Participation**	**Organization/ Hospital environment**	**Professional Attitude**
**Overall experience**					
**Q39**	0.44	0.61	0.52	0.37	0.59
**Q40**	0.43	0.64	0.52	0.4	0.59
**Overall impressionn**					
**Q41**	0.49	0.6	0.46	0.41	0.62
**Q42**	0.47	0.57	0.46	0.39	0.63

*all correlations were statistically significant; p<0.001

[a] answers options were the same as Q1-Q38 items.
[b] answers options were 'very dissatisfied/quite dissatisfied/neither satisfied nor dissatisfied/quite satisfied/very satisfied'.

Supplementary Table 6. Multiple regression results for associations between domain scores and NICU levels, models were adjusted age, level of education, residence, occupation, length of stay. Missing observations were deleted listwise.

Outcome		***(Intercept)***	***Level I NICU[a]***	***Level III NICU[a]***
Communication	Coeff	5.10	-1.55	-0.88
	SE	0.15	0.12	0.08
	P	< 0.0001	< 0.0001	< 0.0001
Care and Treatment	Coeff	5.29	-0.86	-0.88
	SE	0.15	0.12	0.08
	P	< 0.0001	< 0.0001	< 0.0001
Parental Participation	Coeff	5.22	-0.89	-0.15
	SE	0.15	0.12	0.08
	P	< 0.0001	< 0.0001	0.072

Organization/ Hospital environment	Coeff	5.40	-1.05	-1.17
	SE	0.15	0.12	0.08
	P	< 0.0001	< 0.0001	< 0.0001
Professional Attitude	Coeff	5.39	-1.73	-0.77
	SE	0.15	0.12	0.08
	P	< 0.0001	< 0.0001	< 0.0001
Total satisfaction score	Coeff	5.28	-1.25	-0.78
	SE	0.15	0.12	0.08
	P	< 0.0001	< 0.0001	< 0.0001

[a] Level II NICU is taken as reference level.

Supplementary Table 7. Univariate associations (Fisher exact tests) between participants sociodemographic characteristics and general questions on overall experience and impression.

	**EXPERIENCE**				**IMPRESSION**			
	**Q39, n (%)**		**Q40, n (%)**		**Q41, n (%)**		**Q42, n (%)**	
	**Not at all/small/in some extent/largely**	**Not at all/small/in some extent/largely**	**Not at all/small/in some extent/largely**	**Not at all/small/in some extent/largely**	**Not satisfied/ Neither satisfied nor unsatisfied**	**Not satisfied/ Neither satisfied nor unsatisfied**	**Not satisfied/ Neither satisfied nor unsatisfied**	**Not satisfied/ Neither satisfied nor unsatisfied**
**Language**								
Amharic	49 (17.7)	228 (82.3)	48 (17.3)	229 (82.7)	45 (16.2)	232 (83.8)	48 (17.3)	229 (82.7)
Afan Oromo	12 (11.0)	97 (89.0)	8 (7.3)	101 (92.7)	11 (10.1)	98 (89.9)	21 (19.3)	88 (80.7)
P[a]	0.12		0.01		0.15		0.66	
**Age (26 years is the sample median age)**								
≤26 years	34 (17.1)	165 (82.9)	31 (15.6)	168 (84.4)	27 (13.6)	172 (86.4)	33 (16.6)	166 (83.4)
>26 years	27 (14.4)	160 (85.6)	25 (13.4)	162 (86.6)	29 (15.5)	158 (84.5)	36 (19.3)	151 (80.7)
P[a]	0.49		0.57		0.66		0.51	
**Education's level**								
No education	7 (9.2)	69 (90.8)	5 (6.6)	71 (93.4)	4 (5.3)	72 (94.7)	6 (7.9)	70 (92.1)
Low education	12 (9.0)	122 (91.0)	10 (7.5)	124 (92.5)	11 (8.2)	123 (91.8)	15 (11.2)	119 (88.8)
High education	42 (23.9)	134 (76.1)	41 (23.3)	135 (76.7)	41 (23.3)	135 (76.7)	48 (27.3)	128 (72.7)
P[a]	<0.001		<0.001		<0.001		<0.001	
**Occupation**								

Homeworking	31 (12.5)	217 (87.5)	27 (10.9)	221 (89.1)	24 (9.7)	224 (90.3)	30 (12.1)	218 (87.9)
Others	30 (21.7)	108 (78.3)	29 (21.0)	109 (79.0)	32 (23.2)	106 (76.8)	39 (28.3)	99 (71.7)
P[a]	0.02		0.01		<0.001		<0.001	
**Length of stay**								
<1 week	30 (13.0)	201 (87.0)	29 (12.6)	202 (87.4)	32 (13.9)	199 (86.1)	38 (16.5)	193 (83.5)
1-2 weeks	20 (17.1)	97 (82.9)	18 (15.4)	99 (84.6)	17 (14.5)	100 (85.5)	23 (19.7)	94 (80.3)
>2 weeks	11 (28.9)	27 (71.1)	9 (23.7)	29 (76.3)	7 (18.4)	31 (81.6)	8 (21.1)	30 (78.9)
P[a]	0.05		0.18		0.69		0.62	
**Residence**								
Rural	16 (12.5)	112 (87.5)	11 (8.6)	117 (91.4)	13 (10.2)	115 (89.8)	20 (15.6)	108 (84.4)
Urban	44 (17.5)	208 (82.5)	44 (17.5)	208 (82.5)	43 (17.1)	209 (82.9)	49 (19.4)	203 (80.6)
P[a]	0.24		0.02		0.09		0.4	

[a] Fisher's exact tests and Wilcoxon rank-sum test.

Supplementary Table 8. Logistic Regression odds ratios [a] (OR) and 95% confidence intervals results estimating difference for general experience and impression in satisfaction agreement between participants sociodemographic characteristics.

	**EXPERIENCE**						**IMPRESSION**					
	Q39			Q40			Q41			Q42		
	OR[a]	95 %CI	P	OR[a]	95 %CI	P	OR[a]	95 %CI	P	OR[a]	95 %CI	P
**Level I NICU**	0.03	[0.00, 0.11]	<0.001	0.05	[0.01, 0.22]	<0.001	0.26	[0.07, 1.00]	0.044	0.14	[0.05, 0.37]	<0.001
**Level III NICU**	0.07	[0.01, 0.25]	<0.001	0.09	[0.01, 0.30]	0.001	0.27	[0.09, 0.67]	0.008	0.55	[0.24, 1.17]	0.13

[a] Models were adjusted for age, education's level, occupation, child's length of stay and residence area.
